# The Effect of Physical Activity on Combined Cadmium, Lead, and Mercury Exposure

**DOI:** 10.3390/medsci12040071

**Published:** 2024-12-11

**Authors:** Akua Marfo, Emmanuel Obeng-Gyasi

**Affiliations:** 1 Department of Built Environment, North Carolina A&T State University, Greensboro, NC 27411, USA; 2 Environmental Health and Disease Laboratory, North Carolina A&T State University, Greensboro, NC 27411, USA

**Keywords:** metals, physical activity, mixtures, lead, cadmium, mercury

## Abstract

*Background/Objective*: Environmental exposures, such as heavy metals, can significantly affect physical activity, an important determinant of health. This study explores the effect of physical activity on combined exposure to cadmium, lead, and mercury (metals), using data from the 2013–2014 National Health and Nutrition Examination Survey (NHANES). *Methods:* Physical activity was measured with ActiGraph GT3X+ devices worn continuously for 7 days, while blood samples were analyzed for metal content using inductively coupled plasma mass spectrometry. Descriptive statistics and multivariable linear regression were used to assess the impact of multi-metal exposure on physical activity. Additionally, Bayesian Kernel Machine Regression (BKMR) was applied to explore nonlinear and interactive effects of metal exposures on physical activity. Using a Gaussian process with a radial basis function kernel, BKMR estimates posterior distributions via Markov Chain Monte Carlo (MCMC) sampling, allowing for robust evaluation of individual and combined exposure-response relationships. Posterior Inclusion Probabilities (PIPs) were calculated to quantify the relative importance of each metal. *Results:* The linear regression analysis revealed positive associations between cadmium and lead exposure and physical activity. BKMR analysis, particularly the PIP, identified lead as the most influential metal in predicting physical activity, followed by cadmium and mercury. These PIP values provide a probabilistic measure of each metal’s importance, offering deeper insights into their relative contributions to the overall exposure effect. The study also uncovered complex relationships between metal exposures and physical activity. In univariate BKMR exposure-response analysis, lead and cadmium generally showed positive associations with physical activity, while mercury exhibited a slightly negative relationship. Bivariate exposure-response analysis further illustrated how the impact of one metal could be influenced by the presence and levels of another, confirming the trends observed in univariate analyses while also demonstrating the complexity varying doses of two metals can have on either increased or decreased physical activity. Additionally, the overall exposure effect analysis across different quantiles revealed that higher levels of combined metal exposures were associated with increased physical activity, though there was greater uncertainty at higher exposure levels as the 95% credible intervals were wider. *Conclusions:* Overall, this study fills a critical gap by investigating the interactive and combined effects of multiple metals on physical activity. The findings underscore the necessity of using advanced methods such as BKMR to capture the complex dynamics of environmental exposures and their impact on human behavior and health outcomes.

## 1. Introduction

### 1.1. Wearable Devices and Physical Activity

Wearable technology, or wearables, comprises gadgets or equipment worn in direct contact with the body or lightly tethered to a person. Wearables can be categorized as primary or secondary. Primary wearables are autonomous, functioning independently and often serving as central connectors for other devices, such as wrist-worn fitness trackers and smartphones. Secondary wearables, on the other hand, capture specific actions or measurements, like heart rate monitors worn around the chest, and offload the data to a primary device for analysis [1]. Wearable technology can be clothes or accessories suitable for wearing on the human body or intrusive variants such as smart tattoos or microchips [2]. They offer diverse monitoring and scanning capabilities incorporating biofeedback and additional sensory physiological functionalities such as biometry-related functionalities [3]. With their ability to continuously track and monitor physiological metrics, wearable technologies provide valuable data that can help deepen our understanding of physical activity and its critical role in promoting health and preventing chronic diseases.

Higher levels of physical activity have been linked to the prevention and delayed onset of numerous noncommunicable chronic conditions, including heart disease, diabetes, and cancer, as well as various health benefits [4]. Regular physical activity can reduce the risk of heart disease, coronary heart disease, and stroke [5]. Physical activity plays a critical role in managing diabetes by improving insulin sensitivity and blood sugar control. Individuals at risk of developing type 2 diabetes can help prevent or delay its onset through regular and consistent physical activity. Physical activity can also help in burning calories and increasing metabolism, which is effective for weight loss and weight maintenance [6].

Wearable devices have been innovated to track, monitor, and analyze the daily physical activities of users. The devices have built-in sensors that track movement, heart rate, and location. The data obtained is processed to give insights into the user’s activity, which includes steps taken, calories burned, and distance traveled. Information about the user can easily be obtained from the wearable device itself or through applications on smartphones. Examples of wearables used to monitor physical activities include smartwatches, fitness trackers, health monitoring implants, etc. [4].

### 1.2. Metals: Lead, Mercury, and Cadmium

According to the Agency for Toxic Substances and Disease Registry (ATSDR, 2013), lead, mercury, and cadmium are ranked as the second, third, and seventh elements, respectively, on the list of substances posing the highest potential threat to human health. These metals are non-threshold toxicants, capable of causing toxic effects even at very low concentrations [7]. Over the years, elevated cadmium, lead, and mercury concentrations across various natural systems—including the atmosphere, soil, water, and living organisms—have become a global concern [8]. These metals have severe adverse effects on microorganisms, plants, and animals. In humans, exposure to these heavy metals can lead to serious health damage and impair bodily functions, with extreme exposures potentially resulting in death [9]. A study conducted by Sanders et al. between 2009 and 2014 concluded that even minimal childhood exposure to metals such as lead, cadmium, and mercury can adversely impact kidney function. These early disruptions in renal health may increase the risk of developing conditions such as hypertension, chronic kidney disease, and renal dysfunction later in life, underscoring the long-term implications of metal exposure on overall health [10]. Lead, mercury, and cadmium are heavy metals widely dispersed across the environment and can harm humans when present at various exposure levels. Persistent exposure to these metals can pose significant risks. Even though these metals occur naturally, human activities can heighten exposure by releasing them into the air, soil, water, and food, significantly elevating the environmental burden [11].

This study concentrates on these three heavy metals known for their toxic effects, persistence, and environmental prevalence. These metals act as threats to human health and exhibit strong associations with physical activity. Lead and cadmium are neurotoxic metals that can adversely affect cognitive abilities and motor skills. Exposure to lead, for example, has been closely linked to reduced fine motor skills and impaired executive function, which affects the central nervous system and limits the ability to carry out physically demanding tasks [12]. Exposure to multiple metals, such as lead, mercury, and cadmium, can amplify their harmful effects. Research indicates that combined exposure leads to more severe cognitive and motor impairments compared to exposure to a single metal. This is primarily due to disruptions in neurotransmitter systems, which result in behavioral and neurological changes that may contribute to reduced levels of physical activity [13].

#### Physical Activity and Metals

The relationship between physical activity and blood lead levels (BLL) is complex and not yet fully understood. Certain types of physical activity, particularly those performed outdoors in areas with potential lead contamination, may increase the risk of lead exposure and result in elevated BLL [14,15]. Conversely, other research suggests that physical activity might lower blood lead levels (BLLs) by enhancing lead excretion through sweat [16,17,18,19].

### 1.3. Problem Statement

The human body has developed in a manner where most of its systems require regular physical activity to achieve and maintain optimal development and function [20]. Consistent physical activity has been found to reduce the risk of over 25 chronic health conditions and premature death by at least 20–30% [21]. Physical activity yields extensive benefits, encompassing enhanced mental well-being, reduced risk of cardiovascular disease, better sleep quality, and decreased likelihood of certain cancers [22].

Even though there is enough evidence to suggest a relationship between physical activity and environmental exposure [20], the literature still lacks information regarding the combined effects of metals on physical activity as measured by wearable devices. This study will expand on the current understanding of environmental exposures and physical activity by investigating the combined effects of metals on physical activity, addressing the crucial gap in research.

## 2. Materials and Methods

### 2.1. Participants for the Study

This study utilized National Health and Nutrition Examination Survey (NHANES) 2013–2014 data. NHANES provides an assessment of the nutritional status and general health of a nationally representative sample of non-institutionalized individuals in the United States. Participants were asked to wear a physical activity monitor for a continuous period of 7 days. The ActiGraph GT3X+ model, produced by ActiGraph, based in Pensacola, FL, USA, was the device used, and it is used to register vertical accelerations as “counts”, which signify the relative intensity of each motion. ActiGraph GT3X+ devices were primarily worn on the non-dominant arm, with placement chosen to minimize the impact of dominant hand activities that might exaggerate activity levels. Most of the devices were placed in this manner, which helped standardize data collection across participants. Data were collected at an 80 Hz sampling rate. NHANES used an open-source algorithm to categorize periods as wake wear, sleep wear, or non-wear, assigning a confidence level to each classification. It extracted features like signal power and orientation changes in 1.5 min windows, assigning wake, sleep, or non-wear categories to each 30 s period. For periods of low motion during the Idle Sleep Mode (ISM), the algorithm approximated these as wake or sleep by copying data from the preceding 10 s to provide a reliable estimate. It also considered neighboring periods to refine classifications for short intervals. Finally, it used orientation changes to reclassify extended periods of sleep or non-wear for improved accuracy in distinguishing sleep from non-wear. This particular model was selected for its capability to capture triaxial measurements, extended battery life, and water resistance. Metal levels in blood were measured using mass spectrometry, a precise and sensitive method for detecting metals.

### 2.2. Assessment of Heavy Metals

After conducting a simple dilution during sample preparation, heavy metals such as lead, cadmium, and total mercury were directly quantified in whole blood samples using inductively coupled plasma mass spectrometry. To ensure homogeneity of cellular components, a small aliquot of whole blood was extracted from a larger mixed sample during the dilution phase. The dilution process involved mixing 1 part blood sample with 1 part deionized water and 48 parts diluent, achieving the required concentration for analysis. The prepared liquid samples were then introduced into the mass spectrometer via an inductively coupled plasma ionization source for ionization and subsequent detection. The National Health and Nutrition Examination Survey (NHANES) data collection adheres to strict ethical guidelines. Written informed consent was obtained from all participants before data collection. The National Center for Health Statistics (NCHS) Research Ethics Review Board (ERB) reviewed and approved the study protocol. All procedures were conducted by the Declaration of Helsinki and other relevant ethical standards to ensure the protection and confidentiality of participant information.

### 2.3. Descriptive Statistics and Linear Regression

In this study, a detailed descriptive statistical analysis was conducted to characterize the study population and assess the distribution of key variables related to demographic factors, metal exposures, and physical activity levels. The descriptive statistics for continuous variables were calculated using means, standard deviations, medians, and ranges, while frequencies and percentages were computed for categorical variables. For the comparison of metal levels across physical activity categories, means and standard errors were used to summarize the data. This approach allowed for a clear and concise presentation of the distribution of variables and the examination of potential associations between physical activity and metal exposures.

A multivariable linear regression was conducted to examine the association between metal exposures (cadmium, lead, and mercury) and physical activity. The regression model included cadmium, lead, and mercury as independent variables, and their coefficients, standard errors (SE), and 95% confidence intervals (CI) were estimated to assess the strength and precision of these associations with physical activity.

Each metal was modeled as a continuous variable, and the regression coefficients represent the change in the outcome variable per unit increase in metal concentration, adjusting for other covariates included in the model. Statistical significance was assessed by examining whether the 95% confidence intervals included zero.

### 2.4. Bayesian Kernel Machine Regression (BKMR)

To effectively capture potential interactions and nonlinear effects among the components of the mixture, the Bayesian Kernel Machine Regression (BKMR) method was utilized to model the association between multi-pollutant mixtures and physical activity. BKMR’s application has been validated in similar studies examining pollutant mixtures, highlighting its effectiveness over linear models by capturing pollutants’ nonlinear, additive, or even interactive effects [23]. BKMR employs Bayesian inference to estimate the model, Y = h(Z) + Xβ + ϵ, where Y represents the health outcome, Z is the matrix of exposures, X is a matrix of covariates, β is a vector of regression coefficients, and ϵ is the error term, addressing uncertainties related to the estimation of a high-dimensional set of exposures and multiple-testing corrections [24].

In brief, BKMR uses a Gaussian process model with a radial basis function (RBF) kernel to flexibly model nonlinear relationships and applies spike-and-slab priors to the pollutant components to estimate their individual contributions. RBFs are widely used in kernel methods because they can capture the nonlinear relationships typical in environmental exposure data. BKMR leverages the Gaussian kernel to model such interactions, effectively estimating joint effects of multiple pollutants. The choice of RBFs is supported by their adaptability in addressing complex, high-dimensional data, as highlighted in studies on machine learning and exposure-response modeling [23]. Posterior estimation is performed using Markov Chain Monte Carlo (MCMC) sampling. To assess the contribution of individual pollutants to the overall health effects of the mixture, the recommendations of the BKMR were followed by authors using two approaches: (1) examining the marginal nonlinear concentration-response (C-R) curve between each pollutant and the health outcome while holding the effects of other mixture components constant at the 25th, 50th, and 75th percentiles, and (2) calculating the posterior inclusion probability (PIP), which reflects the likelihood that a specific pollutant was included in the model based on the spike-and-slab variable selection in the posterior sample. PIP and exposure-response interpretations using BKMR have been applied in research on multi-pollutant exposures, showing reliable alignment with observed health impacts in cases of high-dimensional exposures [25]. In this study, all analyses were adjusted for age, gender, income, alcohol consumption, smoking, and physical disability status.

## 3. Results

### 3.1. Characteristics of the Sample Population

The study (Table 1) was made up of a total of 10,175 participants, with 5003 (49.17%) being males and 5172 (50.83%) being females. The mean age was 31.48 years, the maximum age was 80 years, and the median was 26 years. The race/ethnicity of the study group included 17.9% of Mexican Americans, 9.9% of other Hispanic, 37.9% non-Hispanic White, 23.4% of non-Hispanic Black, and 11.1% other races, including multi-racial. Out of the total participants, 42.19% had smoked at least more than 100 cigarettes in life, while 57.78% had not smoked at least more than 100 cigarettes in life. From the study, 69.91% had at least 12 alcoholic drinks in the last year, and 29.94% had not taken at least 12 alcoholic drinks in one year.

Table 2 presents descriptive statistics of the metals of interest. Results revealed lead had the highest mean and median levels within the population of interest.

Physical activity (Table 3) was divided into high and low Physical activity was divided into high and low based on the distribution of the database, with the top 25 percent distribution within the database considered high. Mean levels of metals were assessed within this stratification of physical activity (Table 3).

### 3.2. Correlation Between Environmental Contaminants

**Figure 1:**
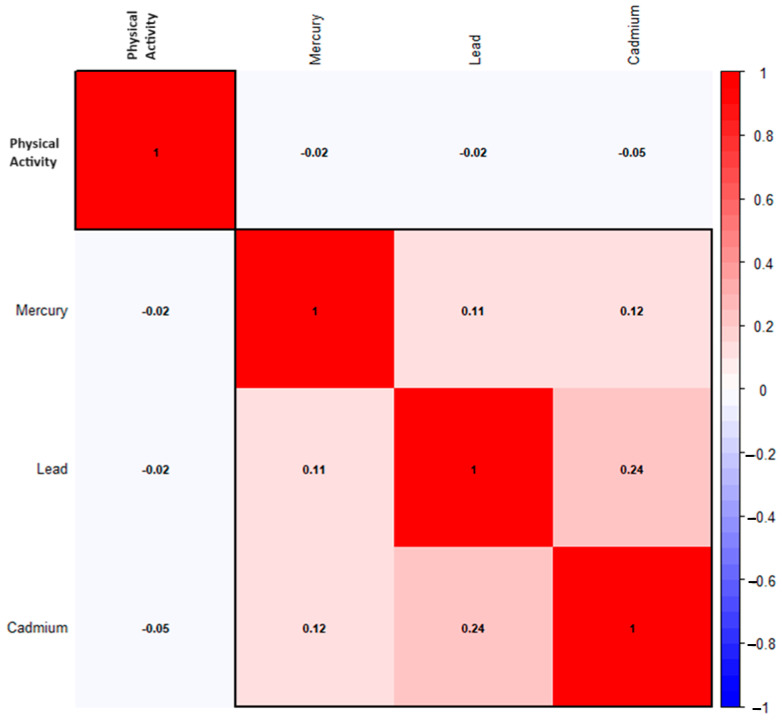
Spearman correlations among physical activity, PFAS, and metals variables. Dark red indicates a strong positive correlation, while dark blue indicates a strong negative correlation.

Figure 1 illustrates the Spearman correlation matrix, which reveals the relationships between various environmental contaminants, including lead, cadmium, and mercury. Of all the relationships examined, lead and cadmium shared the strongest.

Table 4 presents the multivariable linear regression analysis results, assessing the association between physical activity and metal exposures (cadmium, lead, and mercury). The regression coefficients represent the change in physical activity per unit increase in metal concentration, adjusting for relevant covariates.

### 3.3. Bayesian Kernel Machine Regression (BKMR) Analysis

The study explored the complex relationships between the combined effects of metals on physical activity. Traditional linear regression methods, which assume simple linear relationships between variables, often overlook the intricate dynamics present in real-world exposure data. In contrast, Bayesian Kernel Machine Regression (BKMR) leverages kernel functions and Bayesian inference to identify nonlinear and non-additive interactions, offering greater depth and accuracy than linear models.

By employing BKMR, the analysis uncovered insights that traditional linear methods might miss, particularly in addressing the potential nonlinearity and interactions between metals and physical activity. This approach allowed for a more thorough investigation of the underlying mechanisms and the development of new hypotheses.

#### 3.3.1. Posterior Inclusion Probability of Environmental Contaminants with Physical Activity

**Figure 2:**
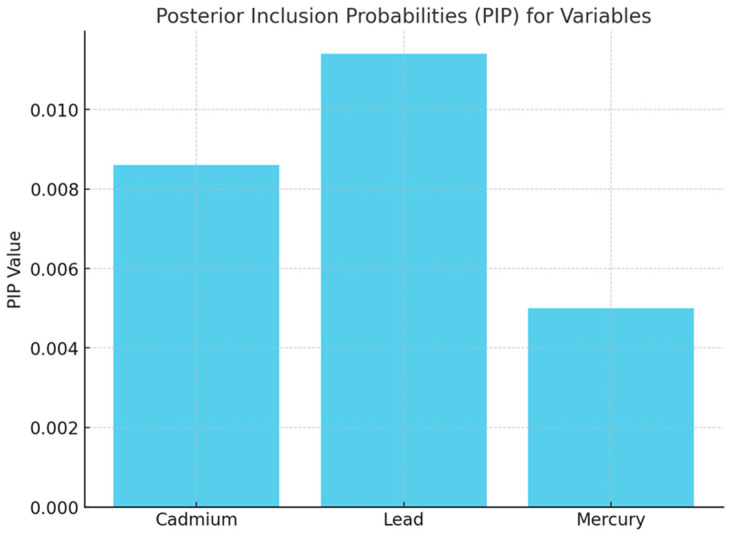
Posterior inclusion probabilities (PIP) for variables. The results revealed that lead had the highest PIP, followed by cadmium and mercury.

Figure 2 shows the BKMR analysis that provided posterior inclusion probabilities to assess the relative importance of each metal (cadmium, lead, and mercury) in predicting physical activity.

#### 3.3.2. Univariate Exposure–Response Relationship of Metals with Physical Activity

**Figure 3:**
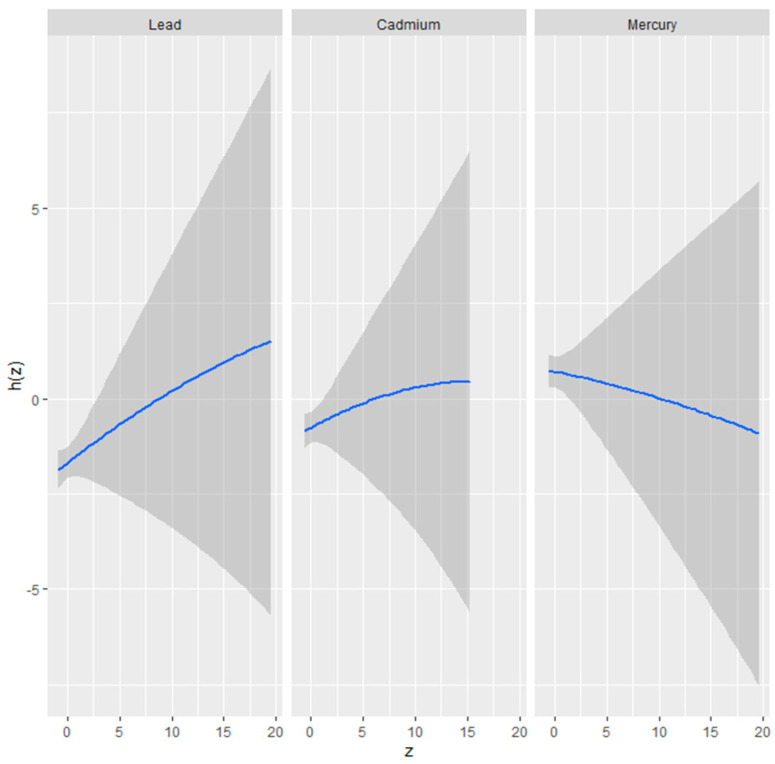
Univariate exposure-response function and 95% credible interval scores for the associations between single pollutant exposures when other pollutant exposures are fixed at the median level. Results adjusted for age, gender, income, alcohol consumption, smoking, and physical disability status. The blue lines show the estimated relationship between each metal and physical activity, while the gray shaded areas represent the 95% credible intervals.

Figure 3 presents univariate exposure-response functions estimated using BKMR for three metals: lead, cadmium, and mercury. The blue lines represent the estimated largely linear relationship between each metal (z) and the outcome variable, while the shaded gray areas indicate the 95% credible intervals, which reflect the uncertainty in the estimated functions. Lead: The exposure-response curve for lead shows a largely linear and slight positive association with the outcome variable. As the concentration of lead increases, the function h(z) gradually increases, suggesting a potential positive relationship between lead exposure and physical activity. Cadmium: The curve for cadmium also shows a largely linear and positive association with the outcome. The slope is similar to that of lead, with an increase in cadmium exposure associated with a modest increase in physical activity. Mercury: The exposure-response curve for mercury exhibits a slightly negative, largely linear relationship.

**Figure 4:**
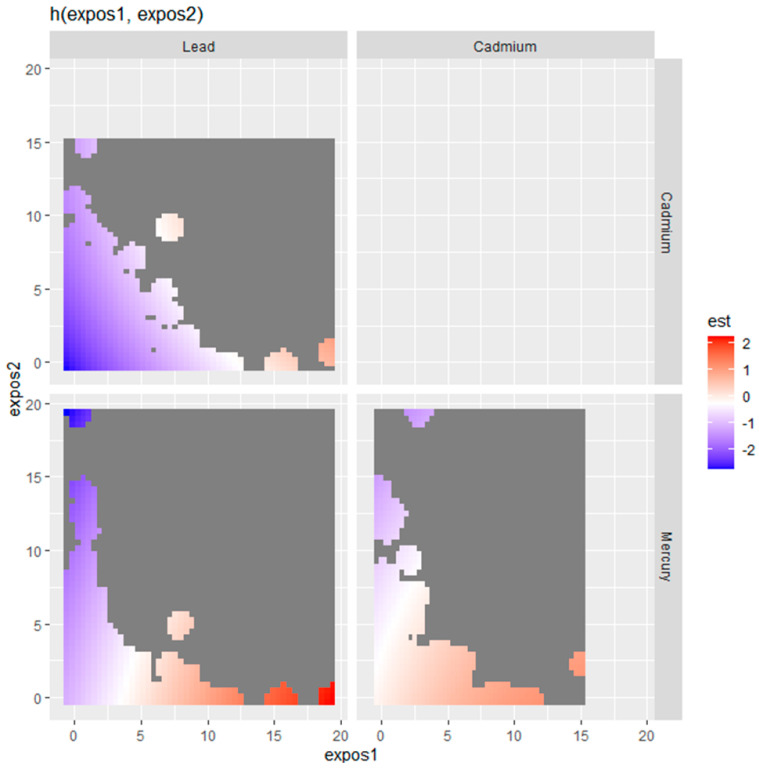
Bivariate exposure–response function of metals with physical activity. Results adjusted for age, gender, income, alcohol consumption, smoking, and physical disability status.

Figure 4 presents bivariate exposure-response functions generated using BKMR to examine the joint effects of pairs of metals (lead, cadmium, and mercury) on physical activity. The colors represent the estimated effects, with red indicating a positive association and blue indicating a negative association. The gray areas suggest regions where the model did not have sufficient data to make reliable estimates. Lead and cadmium: The top-left plot shows the interaction between lead (expos1) and cadmium (expos2) on physical activity. The effect is mostly neutral to slightly negative (indicated by light blue to white), with the strongest positive association occurring at high levels of lead and low levels of cadmium. Lead and mercury: The bottom-left plot illustrates the interaction between lead (expos1) and mercury (expos2). The blue areas at higher levels of lead and mercury indicate a negative association with physical activity at lower exposure levels, meaning that simultaneous exposure to high levels of lead and mercury might reduce physical activity. However, the analysis suggests a positive association between physical activity and higher levels of lead, particularly when mercury levels are low. This pattern indicates that physical activity levels may increase at very high concentrations of lead when accompanied by low mercury exposure. Cadmium and mercury: The bottom-right plot shows the interaction between cadmium (expos1) and mercury (expos2). The plot predominantly shows red shading, indicating a positive association between combined metal exposures and physical activity. However, at low levels of cadmium and high levels of mercury, a shift toward a negative relationship is observed. This suggests that the interaction between cadmium and mercury may influence physical activity in opposing directions under specific exposure conditions. These plots demonstrate the complex interaction between these metals and how varying exposure levels may affect physical activity levels.

**Figure 5:**
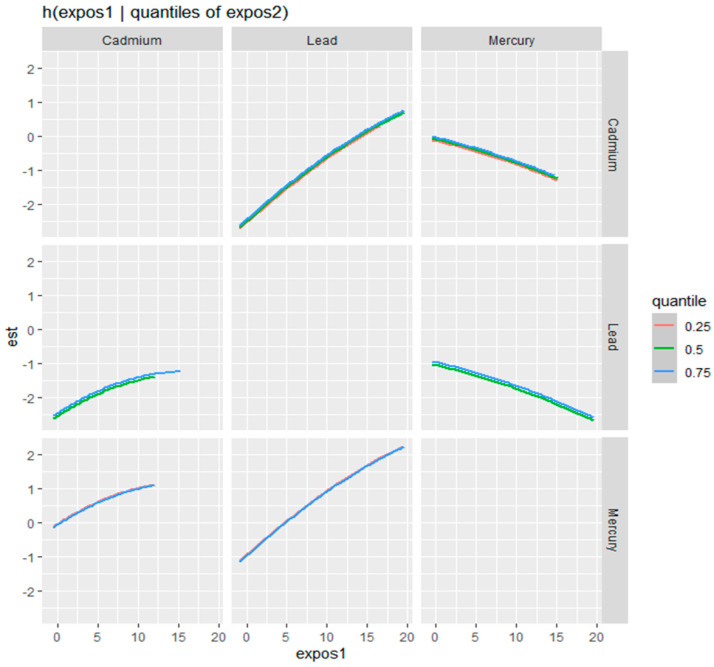
Bivariate exposure–response function of metals with the physical activity—investigating the predictor–response function with varying quantiles of the second predictor while other predictors are fixed. Results adjusted for age, gender, income, alcohol consumption, smoking, and physical disability status.

Figure 5 illustrates the bivariate exposure–response function for three metals (cadmium, lead, and mercury) in relation to physical activity. The figure explores how the predictor–response relationship varies with different quantiles of a second metal exposure (shown in different colors), while controlling for other variables. The *y*-axis represents the estimated effect (est) of the first metal on physical activity, and the *x*-axis represents the level of the first metal exposure (expos1).

Each subplot represents a specific pair of metals, with the response function of the first metal displayed across varying quantiles (0.25, 0.5, and 0.75) of the second metal, indicated by different color lines. For example, in the first column, the response function of cadmium is shown across quantiles of lead and mercury, while in the second column, the response function of lead is shown across quantiles of cadmium and mercury.

The results presented are adjusted for potential confounding factors, including age, gender, income, alcohol consumption, smoking, and physical disability status. This adjustment allows for a more accurate representation of the relationship between metal exposure and physical activity by accounting for other influences.

This figure demonstrates the complexity of the exposure-response relationship and highlights how the effects of one metal may vary depending on the level of exposure to another metal.

#### 3.3.3. Overall Exposure Effect of Combined Metal Exposure on Physical Activity

**Figure 6:**
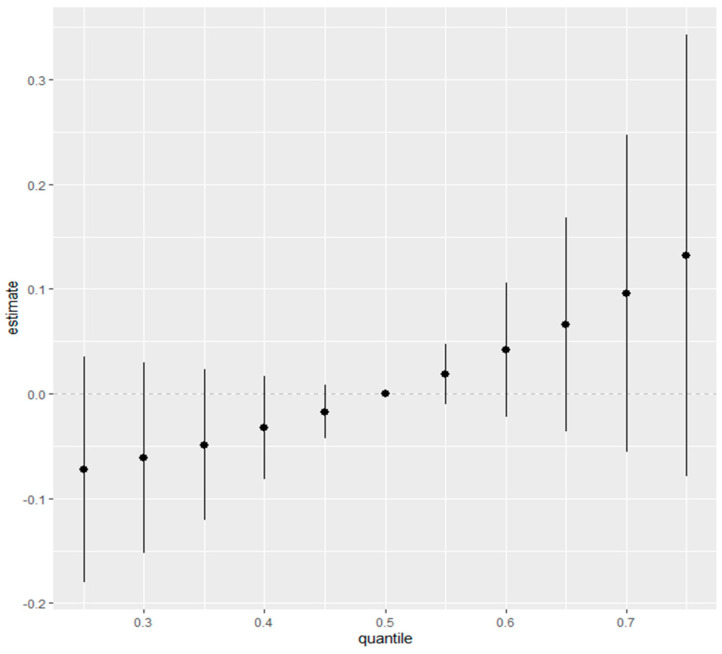
Summary of the overall health effects of the combined metals exposures on physical activity at various quantiles (from 25th to 75th). Results adjusted for age, gender, income, alcohol consumption, smoking, and physical disability status.

Figure 6 represents the overall exposure effect derived from the BKMR model, illustrating how the estimated impact of combined metal exposures varies across different quantiles of the exposure distribution. The *y*-axis indicates the estimated effect on the outcome variable, physical activity, while the *x*-axis denotes the quantiles of the exposure mixture. The general trend observed in the plot is an upward slope, suggesting that as the quantile of exposure increases, the estimated effect on physical activity becomes more positive. This pattern implies that higher levels of combined metal exposures are associated with higher levels of physical activity. The vertical bars on the plot represent the 95% credible intervals, illustrating the uncertainty surrounding the effect estimates. These intervals widen at higher quantiles, indicating greater uncertainty in the effect of high exposure levels on physical activity. Figure 6 suggests a positive trend in the relationship between combined metal exposures and physical activity. However, the credible intervals crossing zero at all quantiles indicate substantial uncertainty in the posterior estimates, suggesting that the data do not provide strong evidence for a consistent association. Within the Bayesian framework, this reflects variability in the estimated effect sizes and highlights the importance of cautious interpretation, as the posterior distribution captures a range of plausible values rather than definitive proof of a relationship. In Figure 6, the *x*-axis (“quantile”) represents the quantiles of all predictors fixed at specific percentiles (e.g., 25th to 75th), while the *y*-axis (“estimate”) indicates the overall estimated effect of the predictors on the outcome (depression) relative to the reference level (50th percentile).

**Figure 7:**
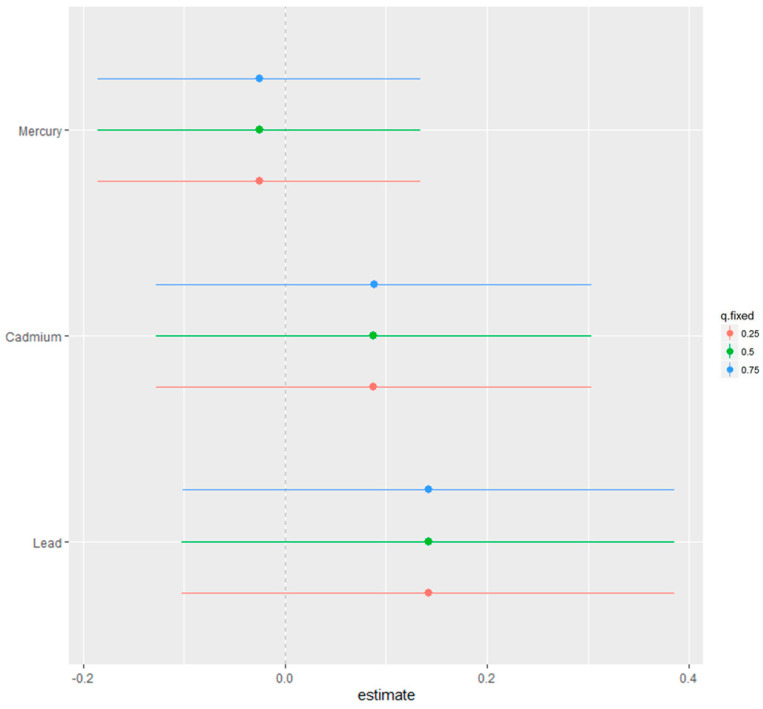
Single variable effect analysis. The changes in outcome are shown as estimates and 95% credible intervals with the single metals level from the 25th to 75th percentiles when all other metals were fixed at their 25th, 50th, or 75th percentiles.

Figure 7 illustrates the results of a single-variable effect analysis using BKMR, focusing on the impact of individual metals (cadmium, lead, and mercury) on physical activity. The analysis examines how the outcome variable, physical activity, changes as the level of a single metal varies from the 25th to the 75th percentiles while simultaneously holding the levels of all other metals together fixed at the 25th, 50th, or 75th percentiles.

This figure shows the estimated effects, along with 95% credible intervals, for each metal individually. By fixing the concentrations of the other metals, the analysis isolates the metal’s influence, allowing for a clearer understanding of its interaction potential and potential impact on physical activity.

## 4. Discussion

### 4.1. Key Study Findings

Understanding the effects of physical activity on combined heavy metal exposure provides valuable insights into how environmental toxicants influence human health and behavior. In the study, physical activity should not be viewed as a proxy for exercise but rather as daily activity, which is often closely linked to socioeconomic status. Individuals with lower incomes are more likely to be in jobs that demand higher levels of physical exertion, a type of form that physical activity trackers can detect. The findings emphasize the importance of examining the complex interactions between multiple metals, as some, like lead, may have a stronger impact during physical activity than others. In the study, Bayesian Kernel Machine Regression analysis identified lead as having the highest posterior inclusion probability among the metals, indicating it is the most likely to be influenced by physical activity levels. This suggests that lead has a more pronounced association with variations in physical activity compared to cadmium and mercury, which had lower posterior inclusion probabilities. The higher posterior inclusion probability for lead reflects its greater contribution to the predictive model, highlighting its significant role in shaping physical activity outcomes [26].

Lead emerged as the most influential metal in predicting physical activity in the study, likely due to its widespread presence and persistence in the environment. According to a study by Kabir and colleagues conducted between March and April 2007, lead is one of the most toxic heavy metal pollutants [27]. It is commonly found in foods such as root vegetables and leafy greens, which can contribute to higher cumulative lead exposure in humans [28]. Over time, this exposure can lead to significant lead accumulation in the body. Lead’s persistence in the body is particularly harmful. Once absorbed, it accumulates in bones and soft tissues, where it can remain for years, continuously releasing into the bloodstream even after external exposure has ceased [29]. This long-term exposure can result in cognitive and motor impairments, as well as behavioral changes. Compared to other metals like cadmium and mercury, which may not bioaccumulate as persistently, lead’s stronger association with physical activity could be more profound due to its ability to remain in the body and cause ongoing harm.

Lead exposure is often more prevalent among vulnerable populations, such as children, women of reproductive age, [30] and low-income individuals, who may face socioeconomic challenges that impact their physical activity levels. In these situations, higher levels of physical activity may stem not from personal choices but from occupational demands or manual labor, which require individuals to stay physically active despite potential adverse effects.

Studies have shown that elevated lead levels have a distinct and consistent relationship with physical activity metrics, aligning with the BKMR’s findings where lead was most influential. Further, nonlinear exposure-response relationships for metals are also documented, as certain metals may exhibit toxic effects only above certain concentrations, which BKMR’s flexible functions can effectively capture in the analysis.

The study found linear relationships between metal exposures and physical activity. Specifically, lead and cadmium were positively associated with physical activity, while mercury exhibited a negative association, highlighting the complex interplay between these metals and physical activity.

Lead primarily enters the body through inhalation, particularly in areas where industrial emissions and lead-contaminated soil are resuspended into the air due to human activities, keeping at-risk populations continuously exposed [15]. The increased likelihood of inhaling lead, combined with its higher environmental burden in most settings, makes it a more likely source of exposure during physical activity. In addition to inhalation, other sources of cadmium exposure include cigarette smoke, food, and drinking water from pipes with galvanized welds [31]. Human exposure to mercury, on the other hand, mainly occurs through the consumption of marine fish [32]. When lead and cadmium are inhaled, their concentrations in the bloodstream increase. Therefore, engaging in physical activity in environments with elevated atmospheric lead and cadmium may result in higher concentrations of these metals in the bloodstream [15]. In contrast, mercury exposure is primarily dietary, and inhalation is not a significant route of exposure. As a result, physical activity may not substantially increase mercury levels in most populations.

The mean levels of metals, including cadmium, lead, and mercury, between participants classified as having “High Physical Activity (PA)” and “Low Physical Activity (PA)” show minimal differences. For cadmium, the mean levels were 0.322 (SE = 0.018) for the high PA group and 0.327 (SE = 0.020) for the low PA group. Similarly, lead levels were 1.04 (SE = 0.061) for high PA and 1.02 (SE = 0.053) for low PA, while mercury levels were 1.00 (SE = 0.055) for high PA and 1.05 (SE = 0.057) for low PA. When assessed using descriptive statistics, these small differences are statistically insignificant, suggesting that physical activity levels do not strongly influence blood metal concentrations.

However, this analysis may not capture the full complexity of the relationship between physical activity and metal exposure. Focusing on group means overlooks important nuances, such as substantial inter-individual variability that could mask meaningful patterns among subgroups of participants. For example, demographic factors might interact with physical activity to influence metal levels differently within subpopulations. Additionally, the relationship between physical activity and metal levels may not be linear or uniform. High-intensity activity could mobilize stored metals from tissues into the bloodstream differently than low- or moderate-intensity activity, a dynamic that mean-level comparisons cannot reveal.

Moreover, the combined impact of multiple metals, which often occur as mixtures, might not align with single-metal assessments. The interplay of these metals could lead to synergistic or antagonistic effects, further complicating the relationship between physical activity and metal concentrations. Using Bayesian methods, such as BKMR, provides a more nuanced understanding by leveraging the kernel function to model nonlinear relationships and interactions among variables effectively. These approaches consider multi-pollutant mixtures and potential confounders while accounting for complex exposure-outcome dynamics. By focusing on mean differences alone, traditional analyses risk oversimplifying a multifaceted relationship, missing critical insights into how physical activity and environmental exposures influence health outcomes.

The results of the multivariable linear regression analysis, assessing the association between metal exposures (cadmium, lead, and mercury) and physical activity, provide limited evidence of strong relationships. For cadmium, the regression coefficient was 0.212 (SE = 0.147) with a 95% confidence interval (CI) of −0.101 to 0.524, suggesting a modest positive association; however, the wide confidence interval includes zero, indicating the result is not statistically significant. Similarly, lead showed a coefficient of 0.019 (SE = 0.056) with a 95% CI of −0.101 to 0.139, reflecting a very small positive association that also fails to reach statistical significance. Mercury had a coefficient of 0.011 (SE = 0.004) with a 95% CI of −0.046 to 0.071, showing no significant relationship between mercury levels and physical activity.

While these results suggest that the associations between physical activity and metal exposures are weak and not statistically significant in this model, relying solely on linear regression may oversimplify the complexity of these relationships. The results might not fully capture potential nonlinear relationships or interactions between variables, as well as the impact of unmeasured confounders or mediators. For example, it is possible that physical activity influences metal exposure differently at various levels of intensity or that other lifestyle or environmental factors modify these associations.

Advanced modeling approaches, such as BKMR, could provide more nuanced insights by capturing nonlinear relationships and interactions that linear regression cannot address. Additionally, these models can better account for the simultaneous influence of multiple metals and covariates, which is particularly relevant given the often-interdependent nature of environmental exposures. While the current analysis provides an initial understanding, its reliance on linear methods and mean effects may obscure the more complex dynamics underlying the relationship between physical activity and metal exposures.

The Bayesian Kernel Machine Regression analysis, designed to assess exposures’ complex and nonlinear effects, revealed minimum interactions between the metals. Each metal appears to act independently, and there is little or no evidence to indicate that their interaction with each other can have an impact on physical activity. This leads to the conclusion that the relationship is more additive rather than synergistic. An additive relationship means that the total effect of the exposure can be viewed as the sum of the individual impacts of each metal. This finding implies that when assessing the health risks, such as impacts on physical activity, it is important to consider each metal’s contribution separately. Since interactions between metals are minimal, regulators and health professionals can prioritize monitoring individual exposure levels of each metal rather than focusing on complex interactions.

However, the analysis of overall exposure effects across different quantiles demonstrated that higher levels of combined metal exposures are associated with increased physical activity, although the uncertainty in these effects increases at higher exposure levels. Individuals who are more physically active may be inadvertently exposed to higher levels of these metals, particularly if their activities involve outdoor environments, such as athletes or workers in contaminated areas. A pilot study conducted by Reche et al. in May 2019 found that athletes are particularly susceptible to air pollution because their air intake is greater, and they spend long periods training and competing outdoors. From the study, a third of athletes reported that air pollution negatively affected their health and training, with the most common symptoms being breathlessness and irritation of the nose, eyes, or throat [33]. It is important to note that higher levels of physical activity may precede metal exposure, making it challenging to establish clear causal relationships between physical activity and metal exposure due to multiple confounding factors. Nevertheless, these findings suggest a complex relationship where combined metal exposures may have varying impacts on physical activity, potentially influenced by specific exposure levels, the nature of the activity, and the environmental conditions involved.

The study’s finding of increased physical activity associated with higher combined metal exposures can be influenced by underlying socioeconomic factors, which may affect activity levels differently across populations. Individuals from lower socioeconomic backgrounds are more likely to engage in physically demanding occupations (e.g., manual work or construction), often in environments with higher pollutant exposure [34]. These jobs lead to increased physical activity as part of the work rather than an effort to promote health. Low-income populations frequently reside near industrial areas with elevated environmental pollutants, such as metals [35]. Their exposure to these pollutants often interacts with outdoor physical activities, like walking or commuting, driven by limited access to transportation options, and may not represent intentional health-promoting behavior. However, individuals exposed to metals due to their jobs or living conditions may engage in physical activity as part of their daily routines, which does not always indicate a healthy decision. Socioeconomic factors can influence these differences in exposure and activity, making it challenging to interpret physical activity solely as a result of environmental exposure.

The findings have important implications for public health, particularly in the context of environmental exposure intervention strategies. Identifying lead as the most influential metal suggests that strategies should be put in place to take it out of outdoor environments as it has profound negative effects on all systems of the human body, including neurotoxicity, nephrotoxicity, and harmful impacts on the hematological and cardiovascular systems [36].

### 4.2. Credible Intervals

The univariate exposure-response relationships for lead, cadmium, and mercury, as shown in Figure 3, demonstrate the importance of credible intervals in interpreting Bayesian analyses. For lead and cadmium, the response patterns suggest a potential positive association with physical activity, while mercury shows a slightly negative trend. The widening credible intervals at higher exposure levels highlight increased uncertainty in these ranges, likely due to limited data availability. This uncertainty does not imply an absence of effect but rather reflects variability in the data and the need for caution when interpreting results at extreme exposure levels.

Figure 6 further emphasizes the role of credible intervals in understanding the overall combined effects of metals on physical activity. While the general trend suggests a slight positive relationship between combined exposures and physical activity, the overlap of credible intervals with zero across all quantiles indicates considerable uncertainty. This suggests that the data does not provide strong evidence for a definitive combined effect, reflecting the inherent complexity of metal interactions and potential variability in their influence on physical activity.

In Figure 7, which examines the effects of individual metals while fixing other metals at specific quantiles, the credible intervals similarly provide essential context for interpreting the results. Lead and cadmium show positive trends, while mercury trends were slightly negative. However, the credible intervals for all metals include zero, reinforcing the uncertainty around these estimates. This underscores the Bayesian framework’s emphasis on probabilistic interpretation rather than frequentist concepts such as statistical significance.

The credible intervals across all figures underscore the need for careful interpretation and highlight areas where data are insufficient or variability is high. Rather than making binary determinations of effect, credible intervals allow us to account for uncertainty and provide a more nuanced understanding of the potential relationships between metal exposures and physical activity. These findings also point to the need for further research to refine estimates and reduce uncertainty, particularly in underrepresented exposure ranges. While the results may not appear conclusive in a frequentist statistics sense, the trends observed have potential public health implications, especially for populations chronically exposed to environmental stressors. This emphasizes the importance of continued efforts to monitor, mitigate, and better understand the health impacts of heavy metal exposure.

### 4.3. Limitations

While the study provides valuable insights, it also has limitations that should be acknowledged. The study has a cross-sectional design that captures metal exposure and physical activity levels at a single point in time, making it challenging to determine a clear cause-and-effect relationship. To address the limitation of a cross-sectional design, alternative methodologies such as longitudinal studies or mediation analysis using BKMR are recommended. These methods, as demonstrated in studies like those by Devick et al., provide stronger causal inferences by evaluating temporal changes or indirect pathways [37].

Tracking participants across multiple time points would clarify whether shifts in metal exposure levels occur before or after changes in physical activity levels. Another limitation of our study was the lack of information on proximity to metal exposure locations, which restricts our ability to fully assess environmental factors influencing metal exposure and physical activity; this limitation highlights the need for future research to address these gaps.

## 5. Conclusions

This study broadens our knowledge of the effects of physical activity on exposure to combined metals such as lead, cadmium, and mercury. Our study highlights the relationships between metal exposure and physical activity, emphasizing the need for additional research to clarify the mechanisms by which these pollutants impact human health.

Individuals, especially those involved in outdoor physical activities, should be encouraged to implement protective measures. They should be encouraged to engage in physical activity using personal protective equipment, like masks, on days with high pollution levels and avoid activities during peak pollution periods. Research has shown that masks can effectively reduce exposure to harmful airborne pollutants, such as particulate matter (PM2.5), without significantly hindering breathing or exercise performance. A study by the University of Saskatchewan confirmed that using a three-layer cloth face mask does not impair breathing during physical activity, suggesting feasibility for routine use during high pollution periods [38].

Addressing these barriers through public health campaigns and policy support could enhance their implementation and effectiveness. Communities should implement focused outreach and educational initiatives, especially in areas that have experienced high lead levels historically. This may include ongoing water quality monitoring and providing resources for safe home renovation practices. Also, campaigns should be launched to inform the public about the health risks linked to lead exposure and strategies to reduce those risks, particularly for vulnerable groups such as children and pregnant women [38]. Overall, our findings highlight the importance of comprehensive strategies to address the interplay between physical activity and combined metal exposures, as well as their implications for overall health.

## Figures and Tables

**Table 1:** Descriptive statistics for critical variables of the study participants.

Variable	N	Percent
Male	5003	49.17
Female	5172	50.83
Race/Hispanic Origin		
Mexican American	1730	17.9
Other Hispanic	960	9.90
Non-Hispanic White	3674	37.9
Non-Hispanic Black	2267	23.4
Other Race—Including Multi-Racial	1074	11.1
Had at least 12 alcoholic drinks in 1 year		
Yes	3790	69.91
No	1623	29.94
Smoked at least more than 100 cigarettes in life		
Yes	2579	42.19
No	3532	57.78

**Table 2:** Descriptive statistics for metals exposure among study participants.

Variable	N	Mean	Minimum	Maximum	Median
Cadium	5215	0.3069	0.07	7.23	0.15
Lead	5215	1.106	0.07	34.1	0.78
Mercury	5215	1.094	0.2	46.4	0.5

**Table 3:** Mean levels of metals were observed through physical activity.

Variable	Mean	
	High PA (SE)	Low PA (SE)
Cadmium	0.322 (0.18)	0.327 (0.020)
Lead	1.04 (0.061)	1.02 (0.053)
Mercury	1.00 (0.055)	1.05 (0.057)

**Table 4:** Linear regression of the association between physical activity and metals.

Variable	Coef. (SE) *	95% CI
Cadmium	0.212 (0.147)	−0.101–0.524
Lead	0.019 (0.056)	−0.101–0.139
Mercury	0.011 (0.004)	−0.046–0.071

* Adjusted for age, gender, income, alcohol consumption, smoking, and physical disability status.

## Data Availability

The data presented in this study are openly available on the CDC NHANES site at https://wwwn.cdc.gov/nchs/nhanes/continuousnhanes/overview.aspx?BeginYear=2013 (accessed on 1 December 2024).
